# A Pilot Study on Gait Kinematics of Old Women with Bound Feet

**DOI:** 10.1155/2015/589709

**Published:** 2015-02-25

**Authors:** Yan Zhang, Neng Feng, Nanzhi Hu, Yaodong Gu

**Affiliations:** ^1^Faculty of Sports Science, Ningbo University, Ningbo 315211, China; ^2^Rehabilitation Center, Ningbo Ninth Hospital, Ningbo 315020, China; ^3^Department of Gynaecology and Obstetrics, The Affiliated Hospital of Medical School of Ningbo University, Ningbo 315020, China

## Abstract

Foot binding has a long and influential history in China. Little is known about biomechanical changes in gait caused by bound foot. The purpose of this study was to investigate the differences in lower limb kinematics between old women with bound feet and normal feet during walking. Six old women subjects (three with bound feet and three controls with normal feet) volunteered to participate in this study. Video data were recorded with a high speed video camera and analysed in the SIMI motion analysis software. Compared to normal controls, bound feet subjects had faster gait cadence with shorter stride length as well as smaller ankle and knee range of motion (ROM). During preswing phase, ankle remained to be dorsiflexion for bound foot subjects. The data from bound foot group also demonstrated that toe vertical displacement increased continuously during whole swing phase without a minimum toe clearance (MTC). The findings indicate that older women with bound feet exhibit significant differences in gait pattern compared to those with normal feet, which is characterised by disappeared propulsion/push-off and reduced mobility of lower limb segments.

## 1. Introduction

Foot binding, an old Chinese custom, had ever gained great popularity and enduring influence on Chinese history. For over thousands of years, young girls about four to seven years old bounded their second to fifth toes tightly to fraction, suffering great pain to prevent further growth of the feet. The small foot size with an extremely high arch [1, 2] is considered as a “golden lotus,” which represents beauty and is sexually exciting to men [3] and also intends to restrict women in home [4]. The literature documented that this cruel tradition spanned over a millennium in Chinese history. It began in Southern Tang Dynasty, flourished in Song Dynasty, and finally was forbidden in the early 20th century [3]. To date, older women aged 65 or above still live with bound feet in some rural and impoverished areas of China, but only few.

With bound feet, it is not easy for women to perform the basic physical activities such as walking, squatting, or even rising from a chair [5]. It is nearly impossible with respect to some intensive activities such as jumping, running, or dancing. Most of previous studies concerning foot binding had been mainly focusing on characterizing foot deformities [6–8] based on the analysis and subject interview to investigate the prevalence of fall history [5]. None of the works have been able to address the fundamental issue on how bound feet affects locomotion, despite the fact that it is believed that bound foot is desirable because it produces a delicate gait [9, 10]. As reported by Mann et al. [7], any type of congenital or acquired defects in bone or soft tissues of the foot might cause impairments functionally and aesthetically. However, there have been very limited quantifiable data available in a biomechanical view to descript bound foot gait pattern due to social and technical difficulties when dealing with subjects at very old group that is gradually disappearing. This work investigated walking kinematic characteristics of old women with bound feet.

## 2. Method

### 2.1. Participants

Three older women with bound feet and three age-matched old women with normal foot shape participated in this study. All subjects have been explained the experiment process and provided written consent before participation. The subjects were able to follow instructions and to complete the walking task safely for 15 min without other person's assistance. The basic characteristics of all subjects are listed in Table 1.

### 2.2. Measurement

Participants wore their own simple manual cloth shoes. Seven reflective markers were attached on anatomical landmarks of the left leg: midthigh, lateral knee supracondylar, midshank, lateral ankle supracondylar, heel, great toe (P_1_), and fifth metatarsal head (P_2_). After determining the lowest point of the shoe (P_toe_) by P_1_ and P_2_ according to Begg et al. [11], P_1_ was removed and another marker was attached to P_toe_ prior to walking test. Prior to testing, a relaxed standing calibration trial was captured first. Both walking and standing tasks were collected using a high speed video camera system (Photron Fastcam SA3, Photron Ltd., Japan) at a sampling rate of 250 Hz. Video data were further analyzed in SIMI motion analysis software (SIMI, Unterschleissheim, Germany). During the tests, all subjects walked at self-selected speed without assistance on a 9.5 m long indoor hard surface walkway. Six trials were carried out.

### 2.3. Data Analysis

Basic spatiotemporal gait parameters as gait speed, stride length, cadence, and the percentage of stance phase in one gait cycle were included. Kinematic data as ROM and angles of knee and ankle in the sagittal plane were included. Data were time normalised to 0 to 100% of the gait cycle. Statistical analyses were performed using SPSS 17.0 (SPSS Inc., Chicago, IL, USA). Differences in joint angles and gait parameters were compared between two groups using multivariate analyses. Due to potential difference in gait velocity and its effect on joint angles, gait velocity was considered as a covariate. Independent *t*-tests were used to assess the differences between two groups for foot length, age, height, and body mass.

## 3. Result

**Figure 1:**
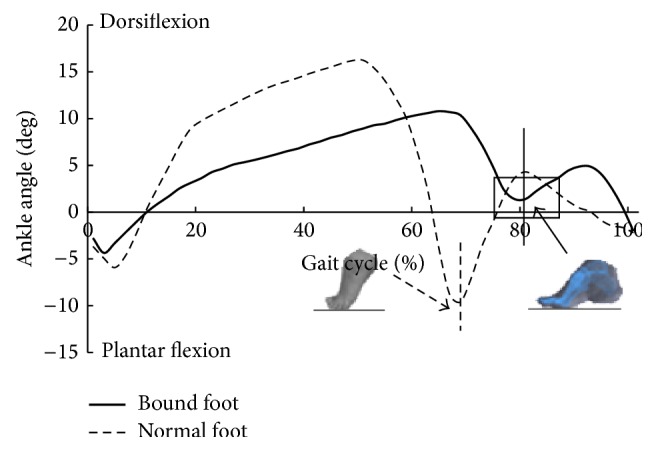
Ankle angle in the sagittal plane during one gait cycle (bold: bound foot group; dashed: control group); vertical line means foot-ground off.

**Figure 2:**
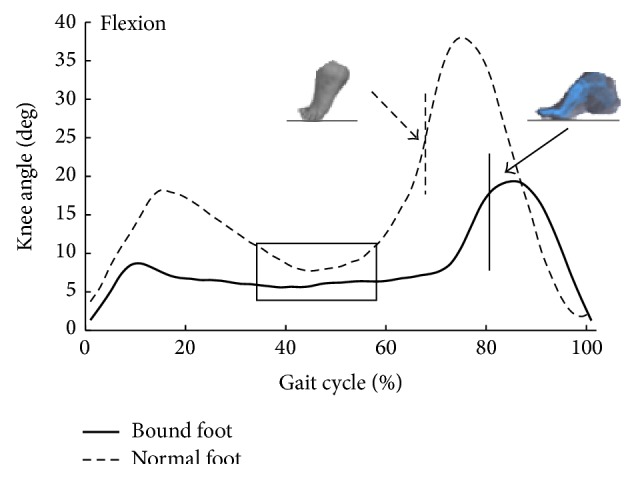
Knee angle in the sagittal plane during one gait cycle (bold: bound foot group; dashed: control group); vertical line means foot-ground off.

**Figure 3:**
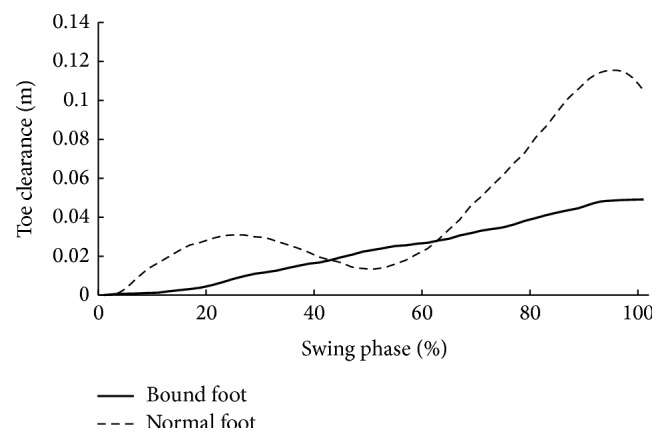
Toe clearance (distance between toes and ground during the swing phase) during swing phase (bold: bound foot group; dashed: control group).

**Figure 4:**
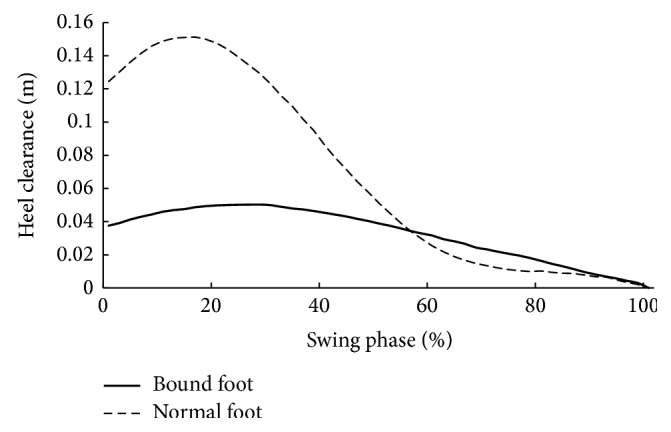
Heel clearance (distance between heel and ground during the swing phase) during swing phase (bold: bound foot group; dashed: control group).

Descriptive statistics for age, height, mass, and foot length are summarized in Table 1. There were no significant differences in age, height, and mass between two groups. However, bound foot subjects had significantly smaller foot length than the control subjects (Table 1).  Table 2 displays spatiotemporal and kinematic parameters of all subjects. Although the average gait speed of normal foot subjects was faster than that of bound foot subjects, it showed no statistical difference between two groups. Moreover, bound foot subjects had a higher cadence with shorter stride length (*P* = 0.046, *P* = 0.037, resp.) compared to the normal foot subjects. The swing phase duration (%GC) of the bound foot was much shorter, only around 20% of one gait cycle, and it was 10% less than the principle percentage. With regard to joint angles, Figures 1 and 2 illustrate typical motions of ankle and knee in the sagittal plane during a whole gait cycle. It was clearly shown that, in comparison with normal foot subjects, those with bound foot performed peculiar motion patterns of lower limb joints. There was a delay of peak dorsiflexion of ankle and an earlier onset of the second knee flexion as follows. Peak angles of ankle dorsiflexion and knee flexion were smaller for bound foot subjects. As well, ROM of ankle and knee in bound foot subjects were significantly smaller (Table 2). Ankle also failed to transfer from dorsiflexion to plantar flexion during preswing phase, and knee had restricted extension during midstance (see the boxes in figures). As to foot motion, an interesting finding was that there was no MTC for bound foot subjects; instead, it increased continuously with a moderate rate throughout swing phase (Figure 3). The total range of toe clearance was smaller than that of the normal. In addition, the vertical displacement of the heel was defined as “heel clearance” in this study; for both groups, it increased during around 20% of swing phase at the beginning and then decreased. However, it showed a milder migration of heel clearance for bound foot subjects, as plotted in Figure 4.

## 4. Discussion

From a cultural perspective, bound foot with ideal foot length of 3 inches symbolises the “golden lotus” [10] which conforms with aesthetic consideration and feudal ideology at that time [12]. However, women had to alter gait pattern due to foot binding. This study demonstrated that the stride length of older women with bound feet decreased significantly while cadence increased significantly compared to those with normal feet. In addition, bound foot subjects walked at a slightly slower self-selected speed but without significance. Therefore, when walking at a similar speed, older women suffering binding deformity took more steps with shorter strides. Similar results were also found in comparison between healthy older women and men, although with normal feet, older women had higher cadence than men [13]. The stance phase of control subjects was close to that reported by Arnold et al. [14]. For bound foot subjects, the increased stance phase in one gait cycle contributes to maintain body balance which was disturbed by shorter foot length and toes fraction.

Differences were also found in foot motion as toe clearance and heel clearance. With bound foot, toe clearance showed a unique trajectory without MTC. This suggested a “pull-off” rather than “push-off” strategy for propulsion in bound foot subjects; as a result, toe clearance in this group increased continuously during whole swing phase. Absence of “push-off” for bound foot women, heel clearance peaked at heel-off and it was consequently lower than normal. However, the time percentages in whole gait cycle where heel clearance peaked for both groups were comparable. This may be associated with increased duration between heel strike and heel-off for bound foot subjects to increase body balance.

Changes in joint angles in the sagittal plane indicate the presence of a less ankle plantar flexion during stance phase and a less mobile lower limb segments in bound foot subjects compared to the controls. The failure of ankle reaching to plantar flexion during preswing phase for bound foot women reinforced the fact that push-off disappeared during walking. This was also consistent with the disappearance of MTC in these subjects. However, energy for forward propulsion is largely relied on ankle power generated during push-off [15]. Previous works have stated that the redistribution of joint power could contribute to forward progression during walking; therefore, the reduced ankle power might be compensated by increased hip power generation during preswing [16–18]. In this case, hip extensors would be strengthened during daily walking, which satisfied men in terms of sexual desire. Another notable result was the longer second knee flexion period for bound foot subjects. This may account for the shorter stride length since earlier onset of knee flexion during preswing limits both knee extension of the ipsilateral leg and further forward swing of the contralateral leg [13]. For bound foot subjects, the second peak knee flexion decreased due to the negation of push-off stage, and the stable knee flexion during midstance might be an adaptive strategy for body control. Meanwhile, bound foot subjects possessed obviously smaller ROM at ankle and knee during whole gait cycle. Thus, foot binding greatly restricts one's ability to participate in social and physical activities through damaging normal supporting structure of the foot.

## 5. Conclusion

In conclusion, foot binding is against natural evaluation, causing adverse effects on one's lower limb biomechanics during walking. This study first unscrambles the ancient Chinese culture, foot binding, in a biomechanical respect. Basically, older women with bound feet had significantly smaller foot length, leading to unique lower limb kinematics in gait compared to those with normal feet. These are primarily characterised by disappeared “push-off” and reduced mobility of lower limb segments. In addition, though increasing cadence, gait speed did not decrease significantly. The findings in this study contribute to the recognition of distinctive changes in foot function in gait for a unique population. Further research is required to establish kinetic changes of this disappearing “golden lotus.”

## Figures and Tables

**Table 1:** Descriptive statistics for age, height, mass, and foot length.

Characteristics for subjects	Bound foot (*N* = 3) mean (SD)	Normal foot (*N* = 3) mean (SD)	*P* value
Age (years)	92.7 ± 1.5	86.7 ± 0.6	0.184
Height (cm)	152.6 ± 1.3	156.5 ± 1.5	0.939
Mass (kg)	48.7 ± 3.3	53.8 ± 2.8	0.774
Foot length (cm)	16.23 ± 1.10	23.23 ± 0.25	**0.045^&^**

SD: standard deviation.

& indicates significance at *P* < 0.05.

**Table 2:** Spatiotemporal and kinematic gait parameters for both groups (mean ± SD).

	Bound foot	Normal foot	*P* value
Spatiotemporal gait parameters			
Gait speed (m/s)	0.64 ± 0.07	0.81 ± 0.11	0.247
Cadence (steps/min)	107.10 ± 4.10	86.30 ± 8.41	**0.046^&^**
Stride length (m)	0.67 ± 0.09	1.04 ± 0.08	**0.037^&^**
Stance (%GC)	80.17 ± 3.94	68.47 ± 5.89	**0.001^&^**
Kinematic gait parameters			
Knee ROM (degree)	18.15 ± 2.06	36.24 ± 4.63	**0.049^&^**
Ankle ROM (degree)	15.36 ± 1.78	25.82 ± 3.65	**0.025^&^**
Knee_swing_ (degree)	18.05 ± 3.12	28.53 ± 2.35	**0.041^&^**
Ankle_swing_ (degree)	1.38 ± 0.53	−9.46 ± 1.34	**0.015^&^**

SD: standard deviation.

& indicates significance at *P* < 0.05.
